# Functional fatty acid-based dietary therapy as a new strategy for small gallstone expulsion: study protocol for a prospective, single-center, double-blind, randomized controlled trial

**DOI:** 10.3389/fnut.2026.1859377

**Published:** 2026-07-03

**Authors:** Long Deng, Han Xu, Bodong Zhang, Kecheng Jin, Ping Yue, Yanyan Lin, Peilun Peng, Emmanuel Melloul, Jinqiu Yuan, Wenbo Meng

**Affiliations:** 1 The First Hospital of Lanzhou University, Lanzhou, Gansu, China; 2 Cardiology Department, Gansu Provincial Central Hospital, Lanzhou, Gansu, China; 3 Lanzhou University, Lanzhou, Gansu, China; 4 University Hospital of Lausanne, Lausanne, Switzerland; 5 Clinical Medical Research Center, The Seventh Affiliated Hospital of Sun Yat-sen University, Shenzhen, Guangdong, China

**Keywords:** cholelithiasis, dietary intervention, functional fatty acids, randomized controlled trial, small gallstones, study protocol

## Abstract

**Background:**

Gallstone disease is a major global health concern with a steadily increasing prevalence, affecting approximately 7% of the global population and imposing substantial health and economic burdens. Globally, gallstone disease accounts for 10–15% of all gastrointestinal hospital admissions, with annual direct medical costs exceeding $6 billion in the United States and ¥20 billion in China, primarily driven by cholecystectomy and post-surgical complications. According to clinical observations, oral administration of olive oil may facilitate the expulsion of small gallstones; however, high-quality evidence supporting this dietary therapy remains limited. In our preliminary study, a 6-day regimen of orally administered functional fatty acids effectively promoted the elimination of small gallstones.

**Objective:**

This study aims to evaluate the efficacy and safety of oral functional fatty acid supplementation for facilitating the expulsion of small gallbladder stones, and to assess its potential as a noninvasive therapeutic option. The primary endpoint is the complete expulsion rate of small gallbladder stones at day 3 post-intervention, confirmed by two independent blinded ultrasound technologists. The complete expulsion rate at day 6 post-intervention is designated as a key secondary endpoint.

**Methods:**

This prospective, single-center, double-blind, randomized controlled trial will enroll 100 adult patients with ultrasonographically confirmed small gallstones (maximum diameter <2 mm) and no prior treatment history. Participants will be randomly assigned at a 1:1 ratio to either the intervention group (*n* = 50), which will receive 50 mL of functional fatty acids daily on an empty stomach for 6 consecutive days, or the control group (*n* = 50), which will receive placebo for the first 3 days followed by the same dose of functional fatty acids for the subsequent 3 days. Abdominal ultrasound examinations will be performed on days 3 and 6 post-intervention to assess gallstone expulsion. Adverse events, including nausea, vomiting, abdominal pain, diarrhea, and signs of biliary obstruction, will be closely monitored throughout the study period.

**Discussion:**

If proven effective, this intervention could provide a noninvasive alternative to surgery for patients with small gallstones, inform clinical practice, and help reduce the healthcare burden associated with gallstone disease.

**Clinical trial registration:**

ClinicalTrials.gov, identifier: NCT06699030

## Introduction

### Background and rationale

Gallstone disease is a significant global health issue (1) that affects approximately 7% of the population, with prevalence increasing steadily in both developed and developing countries (2). This trend has led to substantial health and economic burdens: globally, gallstone disease accounts for 10–15% of all gastrointestinal hospital admissions, with annual direct medical costs exceeding $6 billion in the United States alone and ¥20 billion in China (3–5). These costs are primarily driven by cholecystectomy procedures, which represent the most common abdominal surgery performed worldwide, as well as the management of post-cholecystectomy complications (e.g., persistent abdominal pain, diarrhea, recurrent common bile duct stones) and long-term healthcare utilization (6, 7).

The strategies for treating gallstones are typically categorized into asymptomatic and symptomatic strategies on the basis of their symptoms (8, 9). For asymptomatic gallstones, ursodeoxycholic acid is typically employed to achieve medical litholysis; however, its efficacy is often limited (10, 11). Symptomatic gallstones typically require cholecystectomy, which is generally considered safe but is associated with possible long-term complications, including persistent abdominal pain, diarrhea, and an increased incidence of common bile duct stones (12–14). In addition, surgical interventions are financially and physically burdensome for some patients.

Gallbladder sludge is typically asymptomatic and is most often detected on ultrasound. If left untreated, it can develop into small gallstones measuring less than 2 mm in diameter; in turn, these gallstones can gradually grow and become symptomatic, thus necessitating surgical intervention (15–17). In this study, we define small gallbladder stones as gallstones with a maximum diameter <2 mm, which are also referred to as gallbladder microlithiasis. This term will be used consistently throughout the manuscript to describe our target population.

In our pilot study conducted from April to June 2024 (approved by the Ethics Committee of the First Hospital of Lanzhou University), we enrolled 20 patients with ultrasound-confirmed small gallstones (diameter <2 mm) who received 50 mL of functional fatty acids daily for 6 days. The results showed that 5 (25%) patients achieved complete gallstone expulsion at day 3, and 6 (30%) patients achieved complete expulsion at day 6. No serious adverse events were reported; only 2 patients experienced mild, self-limiting diarrhea that resolved without intervention. The compliance rate (defined as intake of ≥80% of the planned dose) was 100% (18).

Based on these pilot findings, we selected a 6-day intervention duration and established day 3 and day 6 as the primary observation time points, as most stone expulsion events occurred within this window. Additionally, the 25% complete expulsion rate at day 3 in the pilot study was used as the basis for our sample size calculation.

The underlying mechanism may involve greater gastrointestinal motility and increased secretion of cholecystokinin, which promotes gallbladder contraction and facilitates stone expulsion (12, 19–21). However, the limited sample size, absence of a control group, and uncertainty regarding the optimal dose and duration of treatment highlight the need for further investigations to validate these findings.

The aim of this study is to evaluate the efficacy of functional fatty acids as a noninvasive treatment for the expulsion of small gallstones. All participants will provide written informed consent prior to enrollment, and the study will be conducted in accordance with the Consolidated Standards of Reporting Trials (CONSORT) guidelines.

### Aims and objectives

Our aim is to evaluate the efficacy and safety of oral functional fatty acids as a potential nonsurgical approach for promoting the expulsion of small gallstones.

Our specific primary objective is to compare the complete expulsion rate of small gallbladder stones at day 3 post-intervention between the two groups, as confirmed by two independent blinded ultrasound technologists. A key secondary objective is to compare the complete expulsion rate at day 6 post-intervention to evaluate the effect of treatment duration.

In addition, we will determine the incidence and severity of treatment-related adverse events (such as gastrointestinal symptoms and abdominal pain) in the treatment group according to the Common Terminology Criteria for Adverse Events (CTCAE) Version 5.0. In addition, we will evaluate the safety of the oral functional fatty acids by measuring changes in key biochemical markers before and after the intervention, including serum alanine transaminase (ALT), aspartate transaminase (AST), and total cholesterol. ALT and AST are sensitive markers of hepatocellular injury, and their measurement is necessary to assess whether oral administration of large volumes of fatty acids imposes excessive metabolic burden on the liver. Total cholesterol levels will be monitored to detect any adverse effects on lipid metabolism, as well as to evaluate potential changes in bile lipid composition that may contribute to gallstone formation or dissolution.

### Trial design

This is a parallel-group randomized controlled superiority trial with a 1:1 allocation ratio, not a cross-over design. The control group receives placebo for the first 3 days followed by functional fatty acids for the subsequent 3 days. The primary comparisons are: (1) between the experimental group (3 days of functional fatty acids) and the control group (3 days of placebo) at day 3; and (2) between the experimental group (6 days of functional fatty acids) and the control group (3 days of placebo + 3 days of functional fatty acids) at day 6.

This delayed-intervention design was chosen over a 6-day placebo-controlled design primarily for ethical reasons. Our pilot study demonstrated a 25% complete expulsion rate at day 3 with functional fatty acid treatment, and withholding a potentially effective intervention for the full 6 days would be ethically questionable. Additionally, this design allows us to evaluate both the short-term efficacy of the intervention (day 3 comparison: active treatment vs. placebo) and the effect of treatment duration (day 6 comparison: 6 days of continuous treatment vs. 3 days of delayed treatment). Importantly, the day 6 comparison is not between active treatment and placebo, but between two different durations of active treatment.

The core objective of this superiority design is to demonstrate that oral functional fatty acids (experimental intervention) are significantly more effective than the control intervention in promoting the expulsion of small gallbladder stones. This design was selected on the basis of the research hypothesis of the trial: preliminary evidence from preclinical studies and pilot data suggests that compared with the control intervention, functional fatty acids improve gallbladder motility and better regulate bile composition, thereby facilitating the passage of small stones.

The trial is being conducted at the First Hospital of Lanzhou University. The analysis will be based on intention-to-treat (ITT) and per-protocol (PP) methods. A minimum of 50 participants per group is needed to achieve 80% statistical power (with a two-sided *α* = 0.05) in detecting a clinically meaningful difference in the primary efficacy endpoint (e.g., the proportion of participants with complete gallstone expulsion within the treatment period). This sample size calculation is consistent with the superiority design, as it is powered to identify a significant between-group difference in favor of the experimental intervention.

Both participants and researchers will be blinded to the group assignments (double-blinding). The statistician conducting the analysis will remain unaware of the groupings, and the trial nurses will administer the oral treatment. Neither the participants nor the researchers will know which intervention each group receives. Unblinding will be permitted only at the time of statistical analysis, after the trial is completed. In case of an emergency (e.g., acute biliary obstruction), unblinding may be permitted to ensure participant safety. In such an event, the time, reason, and person responsible for the unblinding will be recorded promptly, and the trial monitor will be notified. Participants who are subject to unblinding will be excluded from the final efficacy analysis but will remain in the safety analysis.

In this study, gallstones with a diameter of less than 2 mm were selected as the target objects. The diameter of the cystic duct is generally approximately 2–3 mm, and the inner diameter of the common bile duct is 6–8 mm. Stones smaller than these ductal diameters have the anatomical feasibility to pass through. This selection not only allows the evaluation of gallstone expulsion in the early formation stage (where intervention may be most impactful) but also reduces the potential risk of biliary obstruction—an important safety consideration that aligns with the trial’s goal of assessing both efficacy (per superiority design) and safety.

## Methods

### Primary objective

The primary aim of this study is to determine the effectiveness of oral functional fatty acids in facilitating the expulsion of small gallbladder stones. Upon trial conclusion and researcher and participant unblinding, the pre and postintervention ultrasound examination data will be meticulously analyzed to comprehensively compare the expulsion volume and expulsion rate of gallbladder stones between the experimental group and the control group on the third and sixth days after the intervention through in-depth intergroup comparisons. Moreover, we will perform a detailed analysis of the differences in the expulsion volume and expulsion rate of gallbladder stones within the experimental group to compare the outcomes after a six-day intervention with those after a three-day intervention; relevant data from the control group will be subjected to the same in-depth intergroup analysis.

We hypothesize that the stone expulsion rate among patients in the experimental group who will consume functional fatty acids for 3 days, will be significantly greater than that among patients in the control group who will take placebo over the same period. Additionally, we anticipate that the stone expulsion rate of the experimental group patients who will take functional fatty acids for 6 days will exceed that of the control group patients who will receive placebo supplemented with functional fatty acids. Furthermore, when the experimental group and the control group are compared, the volume of gallstones expelled and the expulsion rate following a six-day intervention are expected to be notably greater than those observed after a three-day intervention.

### Secondary objectives

In this study, relevant indices, including the volume of the gallbladder and the thickness of the gallbladder wall, will be compared among patients via ultrasound examinations before and after the intervention. The purpose of this study is to determine whether the intervention affects gallbladder contraction function and can alleviate gallbladder inflammation.

Moreover, we will compare the rates of clinical adverse events, such as abdominal pain and diarrhea, before and after the intervention. This comprehensive comparison will help us gain a more in-depth understanding of the impacts and safety implications of the dietary therapy studied in this trial.

### Study setting

**Figure 1:**
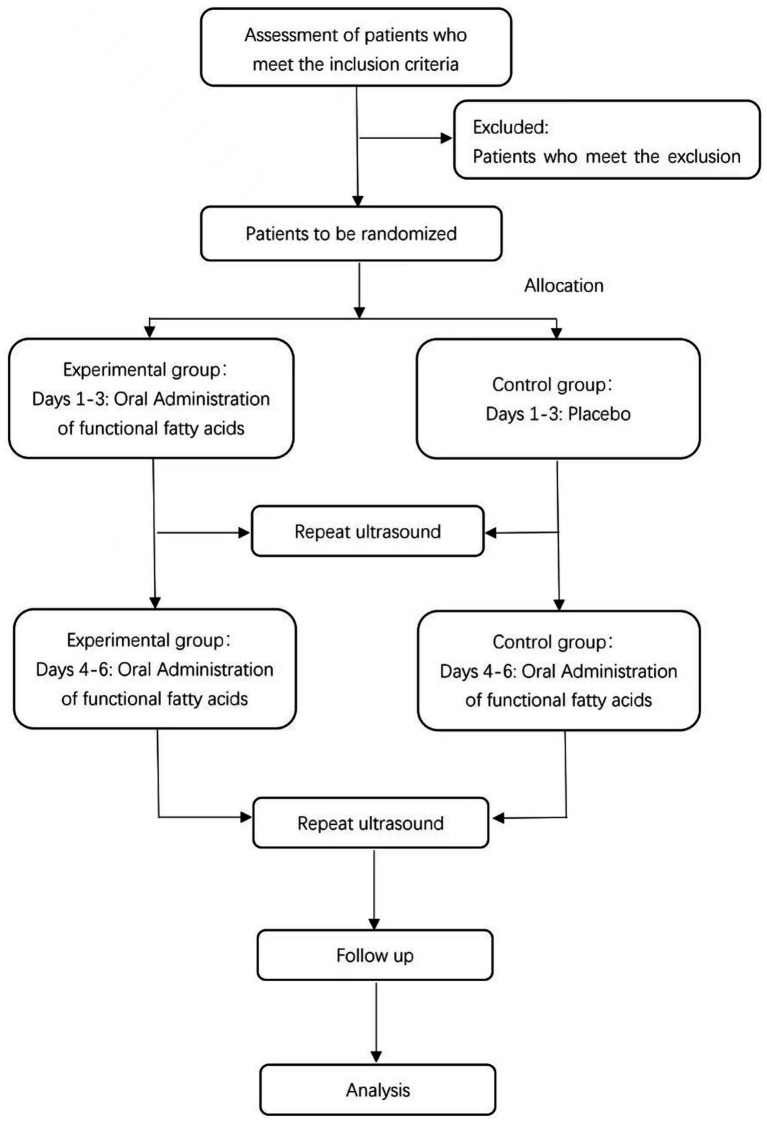
Participant recruitment flow chart.

This trial will be conducted at the Hepatopancreatobiliary Surgery Institute of Gansu Province, located in Lanzhou City, Gansu Province, China. Affiliated with the First Hospital of Lanzhou University, this institute possesses professional expertise in the diagnosis and precision treatment of digestive system diseases, with particularly notable proficiency in the management of biliary tract diseases. An overview of the trial procedure is presented in Figure 1.

### Eligibility criteria

Our target population comprises patients whose electronic medical records revealed small gallstones (defined as gallstones with a diameter less than 2 mm) and who underwent gallbladder ultrasound examinations at this hospital.

Inclusion criteria. (1) Adult patients who were diagnosed with small gallbladder stones (maximum diameter < 2 mm) via ultrasound and had no prior treatment history. (2) Patients of either sex, aged between 18 and 70 years, who were willing to participate in this study.

The exclusion criteria were as follows: (1) age <18 years or >70 years. (2) History of endoscopic retrograde cholangiopancreatography [ERCP]. (3) Previous gallstone removal surgery with gallbladder preservation. (4) History of acute or chronic pancreatitis or cholangitis. (5) Mirizzi syndrome. (6) History of gastrointestinal surgery. (7) Gastrointestinal obstruction. (8) Dysfunction of the sphincter of Oddi. (9) Gallbladder neck polyps. (10) Abnormal gallbladder structure (e.g., the gallbladder has multiple folds). (11) Gallbladder mass. (12) Common bile duct stones, extrahepatic biliary tract stones, or active biliary tract infection. (13) Congenital biliary abnormalities. (14) Biliary injury or surgery. (15) Biliary tumors. (16) Gastrointestinal bleeding, liver cirrhosis, or other malignant diseases. (17) Significant arrhythmia, bradycardia, or atrioventricular block. (18) Severe hypertension or liver or kidney insufficiency. (19) Immune, endocrine, hematological, or mental disorders. (20) Severe cerebrovascular disease. (21) Allergy to relevant foods. (22) Pregnant or breastfeeding women. (23) Unwillingness or inability to consent to participate.

### Intervention description

Data pertaining to the small gallstones will be extracted from the electronic medical records of outpatients who underwent gallbladder ultrasound examination at this hospital. Follow-ups will be conducted via telephone. If a patient meets the inclusion and exclusion criteria, expresses willingness to participate, and has no clinical symptoms related to gallstones, a gallbladder ultrasound will be repeated by a trained and experienced sonographer. Only patients with gallstones measuring less than 2 mm at this follow-up examination will be eligible for enrollment. Eligible patients will be randomly assigned to the experimental group or the control group. The attending physician will explain the potential risks and benefits of the intervention, and written informed consent will be obtained from all participants.

The patients in the experimental group will take 50 mL of oral functional fatty acids on an empty stomach every morning for 6 consecutive days. In the control group, patients will first receive oral placebos for 3 days, followed by the same dose of oral functional fatty acids as the experimental group for the next 3 days. The blinded ultrasound physicians will perform gallbladder ultrasounds on the 3rd and 6th days after treatment to evaluate the effect of small gallstone expulsion. We chose the 3rd and 6th days after the intervention as the observation time points for evaluating the effect of small gallstone expulsion for the following reasons. On the basis of our experience and data from pilot studies, after 6 days of oral functional fatty acids, small gallstones were effectively expelled or exhibited a reduction in their content. However, it remains unclear whether the small gallstones were expelled precisely on the 6th day of intervention or before. Therefore, using the median value as a reference, we aimed to identify an optimal time point for evaluating the expulsion of small gallstones. Well-trained volunteers will follow up with patients during treatment to increase patient compliance and facilitate timely recording and management of any complications.

The functional fatty acids are a proprietary edible oil blend manufactured by Seawit Qingdao Life Science Co., Ltd., China, consisting of 60% medium-chain triglycerides (MCT, predominantly caprylic acid C8:0 and capric acid C10:0) and 40% long-chain triglycerides (LCT, predominantly monounsaturated oleic acid C18:1 and polyunsaturated linoleic acid C18:2). The preparation has a pale yellow color, transparent texture, and light cucumber flavor.

The placebo was specifically formulated to match the functional fatty acids in color, transparency, flavor, and viscosity to maintain blinding integrity. It consists of purified water, 0.15% (w/v) xanthan gum to replicate the oily texture, and cucumber flavoring powder. Trial nurses will prepare the placebo fresh daily immediately before administration to ensure consistent sensory properties.

**Table:** 

Arms	Interventions
One functional fatty acids with a light fruity flavor	Dietary therapy method: Days 1–3: oral administration of functional fatty acids Days 4–6: oral administration of functional fatty acids
Placebo	Days 1–3: oral administration of placebo Days 4–6: oral administration of functional fatty acids

### Outcome measures

#### Primary outcome measures

The primary endpoint is the complete expulsion rate of small gallbladder stones at day 3 post-intervention, confirmed by two independent blinded ultrasound technologists. The complete expulsion rate at day 6 post-intervention is designated as a key secondary endpoint. Complete expulsion of small gallstones is defined as the absence of stone echoes in the gallbladder, with uniform anechoic patterns observed on ultrasound images. When stone echoes persist, multiple diameters of the stones will be measured via ultrasound to estimate their volume, which will then be compared with the preintervention volume to evaluate the partial expulsion amount and rate. The same method will be applied 6 days after intervention.

#### Standardized ultrasound assessment protocol

All ultrasound examinations will be performed using a Philips EPIQ 7 ultrasound system with a 3–5 MHz convex array probe, following a standardized protocol. Patients will be examined in the supine and left lateral decubitus positions after an 8-h fast.

- *Image evaluation*: All images will be independently reviewed by two board-certified sonographers with ≥10 years of experience in abdominal ultrasound, who are blinded to group assignments and previous examination results.
- *Disagreement resolution*: Any discrepancies between the two reviewers will be resolved by a third senior sonographer, whose decision will be final.
- *Image storage*: Both static images (at least 2 images per examination, including longitudinal and transverse views of the gallbladder) and dynamic video clips (5–10 s per view) will be stored in a secure digital repository for future review and quality control.

Measurement standardization:

- *Gallstone number*: Counted in multiple views to ensure all stones are identified
- *Maximum stone diameter*: Measured in three orthogonal planes, with the largest value recorded
- *Gallstone volume*: Calculated using the ellipsoid formula: Volume = (*π*/6) × length × width × depth
- *Gallbladder volume*: Calculated using the ellipsoid formula: Volume = 0.52 × length × width × depth
- *Gallbladder wall thickness*: Measured at the anterior wall of the gallbladder in the longitudinal view, avoiding the neck and fundus regions

#### Secondary outcome measures

Table 1 presents detailed information on the secondary outcome measurements. These data demonstrate the effectiveness and safety of the intervention process, as well as the potential risks.

**Table 1:** Secondary outcome measures.

Secondary outcome measure	Assessment time point	Operational definition
Gallbladder volume	7 days post-intervention	Measured by abdominal ultrasound using the ellipsoid formula: Volume = Length × Width × Depth × 0.52
Gallbladder wall thickness	7 days post-intervention	Measured by abdominal ultrasound at the thickest point, accurate to 0.1 mm
Serum total bilirubin	7 days post-intervention	Determined by standard laboratory assay; normal reference range: 3.4–17.1 μmol/L
Post-intervention abdominal pain	7 days post-intervention	Assessed using the Numerical Rating Scale (NRS)^1^; scores range from 0 (no pain) to 10 (worst imaginable pain)
Post-intervention acute pancreatitis	14 days post-intervention	Defined as the presence of characteristic abdominal pain plus serum amylase level ≥3 times the upper limit of normal^2^
Post-intervention gastrointestinal bleeding	14 days post-intervention	Defined as melena (black tarry stools) or a decrease in hemoglobin (Hb) level ≥20 g/L from baseline^3^
Post-intervention nausea, vomiting, and diarrhea	14 days post-intervention	Recorded as the daily frequency of each symptom during the 14-day follow-up period

^1^NRS: Numerical rating scale. A validated self-report pain assessment tool.

^2^Upper limit of normal for serum amylase at our institution: 100 U/L.

^3^Normal reference range for hemoglobin at our institution: 120–160 g/L (female), 130–175 g/L (male).

^4^All ultrasound examinations will be performed by two independent blinded sonographers with ≥10 years of experience in abdominal imaging. All laboratory tests will be conducted using standard methods at the Clinical Laboratory of the First Hospital of Lanzhou University.

**Figure 2:**
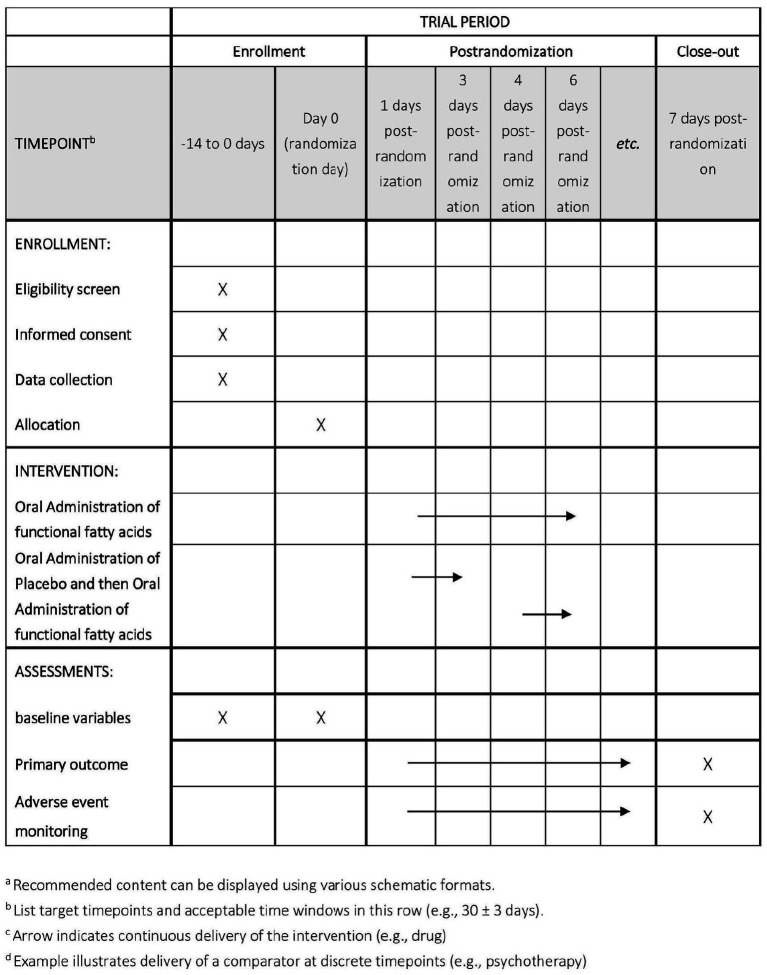
Participant timeline: schedule of enrollment, interventions, and assessments^a^.

Participant timeline please see Figure 2.

### Sample size calculation

In accordance with the ICH E9 Statistical Principles for Clinical Trials, this study is designed to scientifically evaluate the efficacy of functional fatty acids in the removal of small gallbladder stones while optimizing resource efficiency (minimizing cost) and study duration. The sample size calculation is based on rigorous statistical principles to ensure adequate power for detecting clinically meaningful treatment effects, with details as follows:

#### Primary outcome for sample size calculation

The primary outcome used to determine the sample size was the complete expulsion rate of small gallbladder stones at the end of the 6-day intervention period. Stone clearance is defined as the complete disappearance of sedimentary stones confirmed by abdominal ultrasound (the gold-standard imaging method for gallbladder stone assessment in this study), as assessed by two independent ultrasound technologists who are blinded to the treatment assignment. This outcome was selected because it directly reflects the core therapeutic goal of the intervention (i.e., removing small gallbladder stones) and is consistent with primary endpoints used in similar studies of gallbladder stone interventions (22).

#### Assumed outcome rates in the control and intervention groups

Three days after the intervention, the assumed clearance rate of small gallbladder stones in the control group (oral administration of a placebo for 3 days) was 8%. This estimate is supported by published literature on the natural history and placebo response of small gallbladder stones.

Three days after the intervention, the assumed clearance rate of small gallbladder stones in the experimental group (oral administration of functional fatty acids for 3 days, 50 mL/day) was 25%. This estimate is derived from a preliminary study conducted by our team from April to June 2024 (sample size = 20, approved by the Institutional Ethics Review Board: LDYYLL2023-529). In the preliminary study, among the 20 patients who received functional fatty acid treatment, 5 (25%) exhibited complete clearance of small gallbladder stones 3 days after the intervention. No serious adverse events were reported, and the compliance rate (≥80% of the planned dose) was 100%, confirming the feasibility of this intervention.

Therefore, we estimate that the assumed clearance rate of small gallbladder stones in the control group (3 days of placebo + 3 days of functional fatty acids) will still be 25% 6 days after the intervention. However, the assumed clearance rate of small gallbladder stones in the experimental group (oral administration of functional fatty acids for 6 days, 50 mL/day) will be greater than 25% 6 days after the intervention.

#### Allowance for participant attrition

On the basis of historical data from similar gastrointestinal RCTs (23) and our pilot study (attrition rate: 6.25%), we anticipate a 10% attrition rate in the full-scale trial (due to factors such as loss to follow-up, adverse events, or withdrawal of consent). To account for this, we inflated the sample size by 10%. The final sample size per group was calculated as follows: 47 patients (base size) ÷ (1–0.10 attrition rate) ≈ 52 patients.

To simplify randomization and ensure balance between groups, we rounded this number to 50 patients per group, resulting in a total sample size of 100 patients for the study.

### Recruitment

To ensure timely enrollment of the target 100 patients (50 per group) and maintain adherence to the study timeline, a multifaceted, patient-centric recruitment strategy has been designed. This strategy integrates clinical collaboration, targeted outreach, streamlined screening processes, and retention-focused support—all aligned with the study’s inclusion/exclusion criteria (e.g., adults aged 18–70 years with ultrasound-confirmed small gallbladder stones, no prior cholecystectomy, and the ability to complete the 30-day follow-up).

### Allocation

Eligible candidates will be identified through a systematic medical record review and approached for participation via proxy contact. Study randomization will be managed by biostatisticians using a validated computer algorithm to generate a 1:1 allocation sequence, with 100 sequentially numbered sealed opaque envelopes independently encoded and securely stored in a tamper-evident repository.

After confirming that the inclusion criteria have been satisfied, two designated researchers will verify the patient’s identity. They will record the number of unopened envelopes before simultaneously opening the allocation document and signing it together. The trial nurse will administer the corresponding oral treatment protocol to the patient in accordance with their allocation number.

Both the functional fatty acids and the placebo were manufactured by (Seawit) Qingdao Life Science Co., Ltd., China.

Treatment assignments will be securely transmitted by an independent study team. Attending physicians will receive unique assignments for each patient only after all the preintervention protocols have been completed. These protocols include a comprehensive clinical evaluation, the collection of signed informed consent documents, and the confirmation of procedural readiness by the study team.

### Blinding

Both participants and all study personnel involved in intervention administration, clinical assessment, ultrasound examination, and data collection will be blinded to group assignments (double-blinding). The statistician conducting the final data analysis will also remain unaware of group allocations throughout the analysis process. Unblinding will be permitted only after all data have been collected and locked for final statistical analysis.

### Data collection and management

All the data are prospectively collected and managed by well-trained research volunteers.

Baseline data, including age, sex, body mass index (BMI), small gallbladder stone content, gallbladder volume, gallbladder wall thickness, serum bilirubin, the numerical rating scale (NRS) score, postintervention diarrhea, nausea, vomiting, pancreatitis, gastrointestinal bleeding, and other complications, will be collected. This will reflect the characteristics of the participants.

Data, including the expulsion rate of small gallbladder stones and whether there are complications, will be collected each day, starting from 1 day before the intervention to 1 month after the intervention.

Undetected results should have the corresponding symbolic representation and cannot be empty to distinguish them from missing values. During the blind examination period, statisticians should judge the handling of outliers from both medical and statistical aspects.

Ultrasound images, both static and dynamic, will be collected for possible adjunct and long-term studies. In addition, all the patients will be followed up for 1 month after the intervention.

The volunteers who are responsible for collecting the data have extensive clinical experience, are dedicated to the work, and have received training and guidance. Data will be stored by two independent investigators to ensure reliability and validity.

We will also focus on and collect information from participants who stop or deviate before the intervention is completed. To increase participant retention and enthusiasm, we will establish workflow manuals, contact patients regularly, communicate properly and take effective care of their health.

### Statistical analysis

A predefined statistical analysis plan will be followed. All the statistical analyses will be conducted using IBM SPSS (Statistics 30.0.0). A *p* value of less than 0.05 will indicate statistical significance. Categorical variables will be reported as counts and percentages. Continuous variable data will be presented as mean ± standard deviation for normally distributed data, or median (interquartile range, IQR) for non-normally distributed data.

Analyses will be performed on both the intention-to-treat (ITT) population and the per-protocol (PP) population. The ITT population includes all randomized participants who received at least one dose of the intervention. The PP population includes only participants who completed the study protocol without major protocol violations.

The primary analysis will compare the complete expulsion rate of small gallbladder stones at day 3 post-intervention between the two groups using the chi-square test.

Key secondary analyses will include:

1. Comparison of complete expulsion rates at day 6 post-intervention between the two groups
2. Within-group comparisons of changes in gallbladder volume and gallbladder wall thickness from baseline to day 3 and day 6
3. Comparison of the incidence of adverse events between the two groups

For dichotomous variables, the chi-square test or Fisher’s exact test will be used as appropriate. For continuous variables, the independent samples t-test will be used for normally distributed data, and the Mann–Whitney U test will be used for non-normally distributed data.

After the last patient has completed the 30-day follow-up, a blinded endpoint adjudication committee will independently review all primary and secondary endpoint data.

### Data monitoring

Given the excellent safety profile and short duration of this trial, a formal data monitoring committee (DMC) is not required. However, a dedicated safety monitoring team will conduct weekly reviews of all adverse event data throughout the study period.

Although the risk is low, we acknowledge the possibility that small gallstones may become impacted at the sphincter of Oddi, leading to obstructive jaundice or acute biliary pancreatitis. In such cases, the intervention will be immediately discontinued, and the patient will receive appropriate medical treatment according to standard clinical guidelines.

All serious adverse events will be reported to the Institutional Ethics Review Board within 24 h of occurrence. *Ad hoc* safety analyses will be performed if any unexpected safety signals emerge.

### Harms

At each study visit (baseline; weeks 4, 8, and 12), participants will be systematically queried using a standardized checklist covering common gastrointestinal (e.g., nausea and diarrhea) and systemic (e.g., fatigue and headache) events on the basis of the intervention’s known safety profile. Participants will be instructed to report any unexpected symptoms or events (via a 24-h hotline, SMS, or during unscheduled contact) from enrollment until 2 weeks post-intervention.

AEs will be graded using CTCAE v5.0 (mild, moderate, or severe). Investigators will assess the causal relationship to the intervention (unrelated, possibly, probably, definitely related) on the basis of timing and plausibility.

In the case of mild/moderate adverse events, symptomatic treatment (such as antiemetics) may be used if necessary; participants can continue to receive the intervention unless their condition deteriorates.

In the case of severe/relevant adverse events, such as choledocholithiasis and obstructive jaundice, the intervention will be temporarily suspended or terminated, and the participants will be referred to specialists for further treatment.

All AE data will be recorded in the electronic case report form (eCRF) and stored securely. The DSMB will review safety data at week 6 (interim) and week 12 (final) to recommend continuation, modification, or termination of the trial.

### Auditing

An auditing committee consisting of a surgeon and a sonographer will monitor the trial at least quarterly. The committee, independent of the investigators, is responsible for auditing the trial’s frequency, procedures, and safety. All adverse events thought to be related to the trial will be recorded rigorously and carefully. Any unexpected major serious complications suspected to be associated with the intervention will be reported to the interviewer and attending physicians, the trial may be temporarily stopped, and effective treatment measures will be taken by the attending physicians.

### Quality assurance

To enhance the standardization and consistency of this single-center RCT, a comprehensive Standard Operating Procedure (SOP) has been developed that governs all processes from registration, randomization, intervention administration, data collection, and sample management. All the participating personnel, including physicians, nurses, assessors, and trial coordinators, will undergo rigorous SOP training and be required to strictly adhere to the protocol’s guidelines.

### Ethics and dissemination

Each participant provided written informed consent before data collection. The study was approved by the Ethics Committee of the First Hospital of Lanzhou University (LDYYLL-2024-804). Any changes to the eligibility criteria, outcomes, and analyses must obtain approval from the ethics committee. All procedures in the trial will be performed in accordance with the ethical standards of the institutional and/or national research committee and with the 1964 Helsinki declaration and its later amendments or comparable ethical standards.

### Patient and public involvement

Neither the patients nor the public were involved in the design, conduct, reporting, or dissemination plans of this research.

### Confidentiality

Data will be handled confidentially. During the whole experiment, sensitive information such as patient name and identity card number will not be revealed by coding.

### Availability of data and materials

Upon trial completion, the researcher will promptly write and submit the manuscript via open access. Each participant will be mailed the results and conclusions. Owing to patient confidentiality considerations, the raw data from this study are not publicly available. Researchers with a justified need for access may request the data from the corresponding author, subject to appropriate ethical and regulatory approvals.

### Dissemination policy

Data analysis, interpretation, and findings will be presented at academic conferences and published in peer-reviewed journals. As no data from the individuals has been reported within the manuscript, consent for publication of images may not be needed. Neither the patients nor the public were involved in the design, conduct, reporting, or dissemination plans of this research.

## Discussion

To our knowledge, this will be the first prospective, single-center, double-blind, randomized controlled trial to evaluate the therapeutic potential of functional fatty acids in the management of gallbladder calculi. Without timely intervention, small gallbladder stones may progress to form gallstones, leading to a range of health and economic burdens. These include pain-related reductions in quality of life, impaired gallbladder function and the inconvenience and risks associated with surgical removal (24).

To date, several small-scale studies have explored the use of diet or proprietary Chinese medicine for treating gallbladder stones (25). Although some patients in these studies were able to successfully excrete the gallstones, there is a notable lack of evidence-based medical evidence. Consequently, these treatment methods are neither widely promoted nor used in clinical practice. This can be partly due to the limitations of diet therapy, such as the inability to precisely identify gallbladder stone components, the absence of placebo control, and the difficulty of accurately quantifying the diet.

In the pilot study of this research, six patients with sonographically confirmed gallbladder small stones successfully excreted their stones following dietary therapy. The intervention was well tolerated, with only two patients experiencing mild diarrhea after consuming functional fatty acids and no other complications reported. On the basis of these findings, we believe that dietary therapy may hold promise as a treatment option for patients with small gallstones.

A possible biological mechanism underlying the efficacy of functional fatty acids in promoting gallstone expulsion involves multiple complementary pathways:

1. *Stimulation of cholecystokinin (CCK) secretion*: Medium-chain triglycerides (MCTs) are rapidly absorbed in the proximal small intestine, stimulating intestinal I cells to secrete CCK—the primary hormone regulating gallbladder motility. CCK induces strong, coordinated contraction of the gallbladder smooth muscle while simultaneously relaxing the sphincter of Oddi, creating a pressure gradient that facilitates the passage of small stones into the duodenum. Long-chain triglycerides (LCTs) provide a more sustained stimulation of CCK secretion, maintaining gallbladder contractility over an extended period.
2. *Enhanced gastrointestinal motility*: Functional fatty acids promote intestinal peristalsis, which indirectly increases biliary tract motility and accelerates the transit of stones through the common bile duct.
3. *Modulation of bile composition*: Functional fatty acids may alter the lipid profile of bile, reducing cholesterol saturation and inhibiting the further growth of cholesterol stones. In some cases, they may even partially dissolve small cholesterol microliths, making them easier to expel.
4. *Anatomical safety*: The selection of stones <2 mm in diameter ensures anatomical feasibility for passage through the cystic duct (2–3 mm) and common bile duct (6–8 mm), minimizing the risk of biliary obstruction, acute cholangitis, or pancreatitis.

Ultrasound has a high sensitivity for detecting gallstones, but it cannot distinguish between cholesterol and pigment stones solely on the basis of imaging features (26). Therefore, we did not perform relevant analyses on the nature of the stones in this study.
